# Endoplasmic Reticulum Stress-Induced JNK Activation Is a Critical Event Leading to Mitochondria-Mediated Cell Death Caused by β-Lapachone Treatment

**DOI:** 10.1371/journal.pone.0021533

**Published:** 2011-06-29

**Authors:** Hyemi Lee, Moon-Taek Park, Bo-Hwa Choi, Eun-Taex Oh, Min-Jeong Song, Jeonghun Lee, Chulhee Kim, Byung Uk Lim, Heon Joo Park

**Affiliations:** 1 Department of Microbiology, College of Medicine, Center for Advanced Medical Education by BK21 Project, Inha University, Incheon, Republic of Korea; 2 Department of Polymer Science and Engineering, Inha University, Incheon, Korea; Roswell Park Cancer Institute, United States of America

## Abstract

**Background:**

β-lapachone (β-lap) is a bioreductive agent that is activated by the two-electron reductase NAD(P)H quinone oxidoreductase 1 (NQO1). Although β-lap has been reported to induce apoptosis in various cancer types in an NQO1-dependent manner, the signaling pathways by which β-lap causes apoptosis are poorly understood.

**Methodology/Principal Findings:**

β-lap-induced apoptosis and related molecular signaling pathways in NQO1-negative and NQO1-overexpressing MDA-MB-231 cells were investigated. Pharmacological inhibitors or siRNAs against factors involved in β-lap-induced apoptosis were used to clarify the roles played by such factors in β-lap-activated apoptotic signaling pathways. β-lap leads to clonogenic cell death and apoptosis in an NQO1- dependent manner. Treatment of NQO1-overexpressing MDA-MB-231 cells with β-lap causes rapid disruption of mitochondrial membrane potential, nuclear translocation of AIF and Endo G from mitochondria, and subsequent caspase-independent apoptotic cell death. siRNAs targeting AIF and Endo G effectively attenuate β-lap-induced clonogenic and apoptotic cell death. Moreover, β-lap induces cleavage of Bax, which accumulates in mitochondria, coinciding with the observed changes in mitochondria membrane potential. Pretreatment with Salubrinal (Sal), an endoplasmic reticulum (ER) stress inhibitor, efficiently attenuates JNK activation caused by β-lap, and subsequent mitochondria-mediated cell death. In addition, β-lap-induced generation and mitochondrial translocation of cleaved Bax are efficiently blocked by JNK inhibition.

**Conclusions/Significance:**

Our results indicate that β-lap triggers induction of endoplasmic reticulum (ER) stress, thereby leading to JNK activation and mitochondria-mediated apoptosis. The signaling pathways that we revealed in this study may significantly contribute to an improvement of NQO1-directed tumor therapies.

## Introduction

β-lapachone (3,4-dihydro-2,2-dimethyl-2H-naphtho[1,2-b]pyran-5,6-dione; β-lap), a naturally occurring quinone obtained from the bark of the Lapacho tree (Tabebuia vellanedae), has been reported to exert a variety of pharmacological activities, such as anti-bacterial, -fungal, -trypanocidal, and -cancer [1]–[2]. ARQ501 is a formulation of β -lapachone complexed with hydroxypropyl-β-cyclodextrin for the treatment of human tumors [3]. In order to increase the clinical efficacy of β-lap, a novel method to improve intratumoral delivery of β-lap using polymer millirods has been developed [3]. The anti-cancer activity of β-lap was known to be due to two-electron reduction of β-lap mediated by NAD(P)H: quinone oxidoreductase (NQO1, DT-diaphorase) using NADH or NAD(P)H as electron sources [1]–[3]. The NQO1 is expressed abundantly in various human solid cancers, including cancers of the breast, pancreas, lung, and colon [1]–[3]. Therefore, β-lap can selectively kill these human cancer cells that overexpress endogenous NQO1 [4]. Moreover, the futile cycling between the oxidized and reduced forms of β-lap caused by NQO1 leads to progressive depletion of NADH and NAD(P)H, which in turn induces a massive release of Ca^2+^ from Endoplasmic Reticulum (ER) to cytosol, causing activation of calpain known as a Ca^2+^ dependent proteinase and subsequent apoptosis [1], [2], [5].

Mitochondria-mediated apoptotic cell death has been demonstrated to be modulated by Bcl-2 family [6]. Among Bcl-2 family, Bax or Bak performs positive roles in the permeabilization of mitochondrial outer membranes by participating in the formation of pores, facilitating release of cytochrome *c*, apoptosis-inducing factor (AIF), and endonuclease G (Endo G), from the intermembrane space of the mitochondrion to the cytosol. Cytosolic cytochrome *c* binds to apoptotic protease-activating factor 1 (Apaf-1), in a ternary complex with caspase-9, leading to caspase-9 activation; caspase-9 in turn activates caspase-3 [7]. Cleavage of the inhibitor of caspase-activated DNase (ICAD) by caspase-3 results in activation of caspase-activated DNase (CAD), which fragments DNA resulting in apoptotic cell death [7]–[11]. Furthermore, AIF and Endo G released from mitochondria are translocated to the nucleus, within which these molecules trigger large-scale DNA fragmentation, and condense chromatin, leading to apoptotic cell death in a caspase-independent manner [10].

Diverse chemotherapeutic agents have been suggested to cause proteolytic truncation of Bax, and trigger the mitochondrial cell death pathway [12]–[14]. In these reports, Bax is known to be truncated at aspartate 33 by calpain, resulting in the formation of an 18 kDa cleavage product that is more potent than native Bax in terms of stimulating mitochondria-mediated apoptotic cell death [15], [16].

Mitogen-activated protein kinases (MAPKs), which are members of the Ser/Thr protein kinase family, have been demonstrated to be activated in response to a variety of external stimuli, and these kinases participate in the regulation of cell proliferation, differentiation, survival, and apoptosis [17]–[19]. MAPKs can be subdivided into 3 major classes based on sequence homology: extracellular signal-regulated kinases (ERKs), p38 MAPK, and stress-activated/c-Jun N-terminal kinases (JNKs) [19]. ERK is commonly thought to mediate survival when apoptotic stimuli are applied [17], [20]–[22]. In contrast to ERKs, JNK and p38 MAPK respond strongly to a variety of stress signals, including those mediated by cytokines, hyperosmotic stress, ionizing radiation, UV irradiation and chemotherapeutic agents, and cause apoptosis [17], [20]–[22].

The ER is the cellular organelle responsible for biogenesis of proteins and calcium homeostasis [23]–[25]. When unfolded or misfolded proteins are accumulated in ER lumen, cells adopt a protective strategy to overcome this ER stress, termed unfolded protein response (UPR) [23]–[25]. UPR is activated by ER transmembrane proteins, such as inositol-requiring enzyme 1 (IRE1), pancreatic ER kinase (PERK), and activating transcription factor (ATF) [23]–[26]. These proteins play a key role in increasing the expression of various genes including GRP78 and 94 to restore ER homeostasis [23]–[26]. It has been demonstrated that PERK phosphorylates eIF2α (eukaryotic initiation factor 2α), which subsequently activates ATF-4. Thereafter, ATF-4 induces the expression of CHOP (C/EBP-homologous protein) and UPR target genes [27], [28]. Importantly, CHOP and phosphorylated eIF2α have been indicated to be involved in ER stress-induced cell death [27]–[29]. Furthermore, when ER stress is excessive, UPR induces activation of IRE1/ASK1/JNK pathway which leads to apoptotic cell death to remove severely damaged cells [15]–[18].

In this study, we investigated the molecular signaling mechanism leading to apoptotic cell death caused by β-lap in NQO1-overexpressing MDA-MB-231 cells. We demonstrate here that JNK activation due to ER stress significantly contributes to mitochondria-mediated cell death caused by β-lap treatment. Furthermore, our results show that JNK activity is required for generation of cleaved Bax and mitochondrial translocation of cleaved Bax in response to β-lap treatment. Insight gained in the present study on the molecular signaling pathway in β-lap-induced cell death will lead to advances in establishing the strategies for NQO1-targeted tumor treatment.

## Results

### β-lap induces NQO1-dependent and caspase-independent cell death

**Figure 1:**
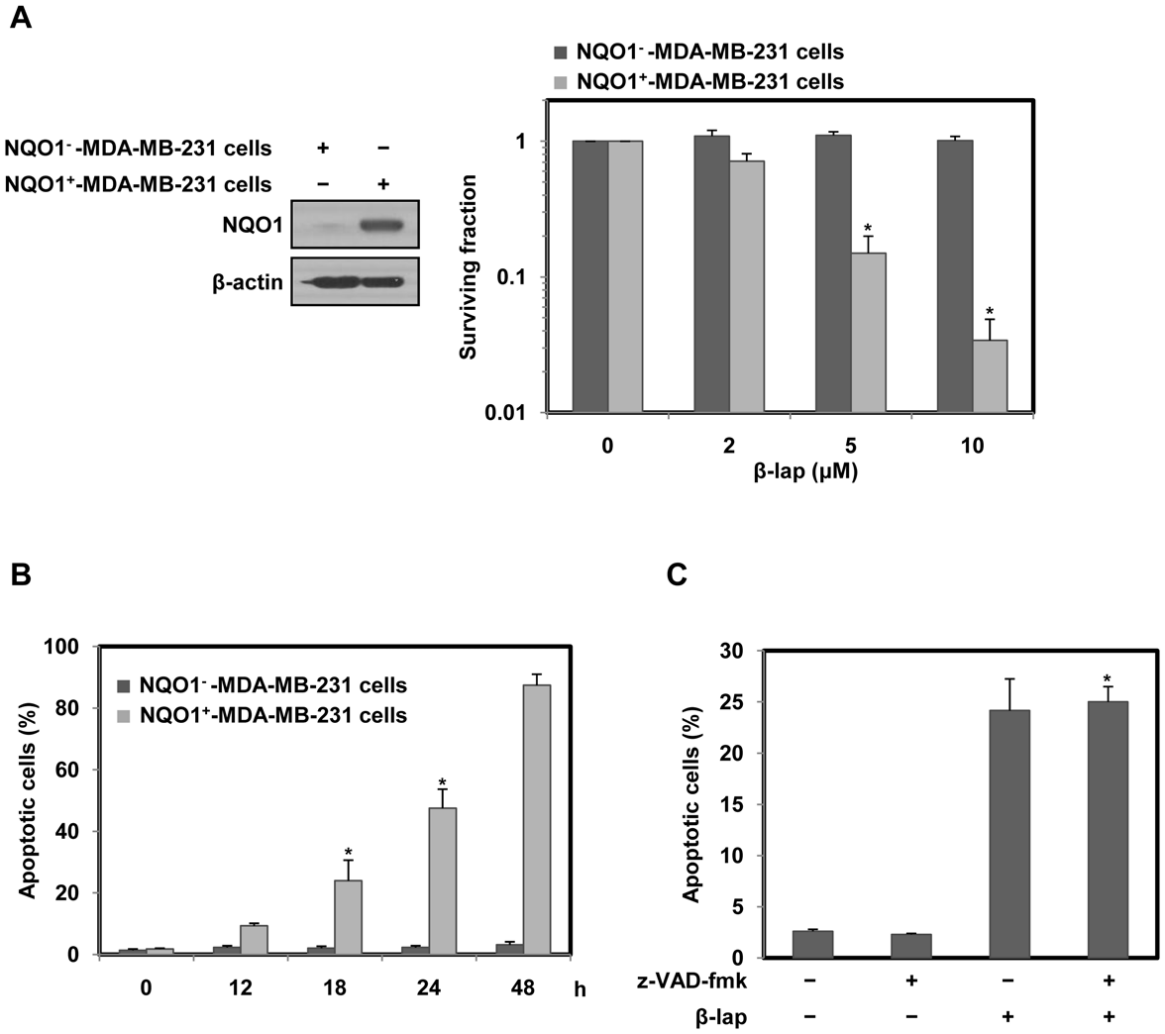
β-lap induces NOQ1-dependnet cell death. (A) NQO1^−^ or NQO1^+^-MDA-MB-231 cells were treated with various concentrations of β-lap. Cells were allowed to grow for 10 to 14 days and were stained with 0.5% crystal violet and scored for colony formation. Results from three independent experiments are expressed as means ± SEM. *Significant difference between NQO1^−^- and NQO1^+^-MDA-MB-231 cells after β-lap treatment at p<0.05. (B) NQO1^−^ or NQO1^+^- MDA-MB-231 cells were treated with 5 µM of β-lap for the indicated times. Cells were stained with PI and the percentage of cells with a sub-G1 DNA content was measured via flow cytometric analysis. Results from three independent experiments are expressed as the means ± SEM. *Significant difference between NQO1^−^- and NQO1^+^-MDA-MB-231 cells after β-lap treatment at p<0.05. (C) NQO1^+^-MDA-MB-231 cells were treated with 5 µM of β-lap for 18 h in the presence or absence of z-VAD-fmk (30 µM). Cells were stained with PI and the percentage of cells with a sub-G1 DNA content was measured via flow cytometric analysis. Results from three independent experiments are expressed as means ± SEM. *Significant difference between the cells in the presence or absence of z-VAD-fmk after β-lap treatment at p<0.05.

To investigate the cell death induced by β-lap, we treated parental NQO1^−^-MDA-MB-231 cells deficient in NQO1, or NQO1^+^-MDA-MB-231 cells possessing abundant expression of NQO1 (Fig. 1A), with different doses of β-lap, and analyzed clonogenic cell death using a clonogenic cell survival assay. Figure 1A shows that a dose-dependent increase in the proportion of clonogenic cell death after β-lap treatment was evident, suggesting that NQO1^+^-MDA-MB-231 cells were more sensitive to β-lap than parental NQO1^−^-MDA-MB-231 cells. To further evaluate whether the cytotoxic effect of β-lap was associated with induction of apoptosis, NQO1^−^- or NQO1^+^-MDA-MB-231 cells were treated with 5 µM of β-lap for various lengths of time, and then analyzed with a sub-G1 DNA content. Figure 1B shows that a time-dependent increase in the proportion of apoptosis after β-lap treatment was apparent in NQO1^+^-MDA-MB-231 cells, but not in NQO1^−^-MDA-MB-231 cells, suggesting that β-lap induces apoptotic cell death in an NQO1-dependent manner.

As the activation of caspase plays a key role in the induction of apoptotic cell death [17], we investigated whether caspase activities are required for β-lap-induced apoptotic cell death in NQO1^+^-MDA-MB-231 cells using a broad-spectrum caspase inhibitor, z-VAD-fmk. This caspase inhibitor was able to prevent activation of caspases (data not shown) but failed to attenuate β-lap-induced apoptotic cell death (Fig. 1C). This result indicates that the apoptotic cell death by β-lap occurs in a caspase-independent manner.

### β-lap induces apoptotic cell death via translocation of AIF and Endo G to the nucleus

**Figure 2:**
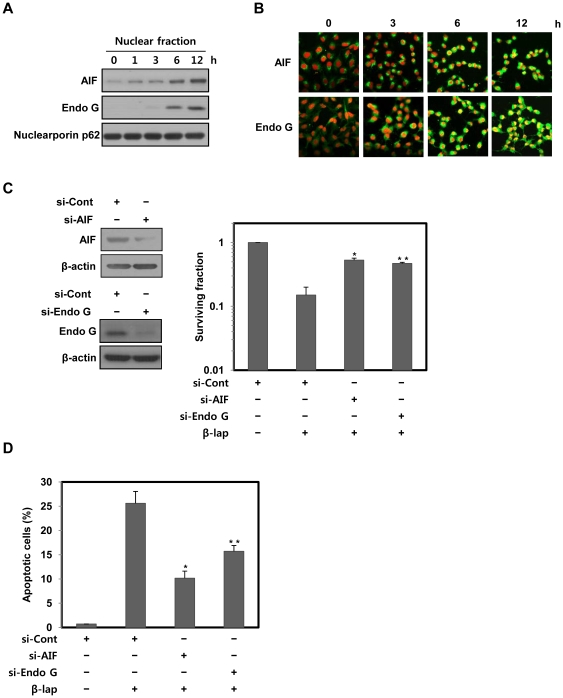
β-lap induces cell death in AIF and Endo G-dependent manners. (A) Nuclear fractions were obtained from NQO1^+^-MDA-MB-231 cells treated with 5 µM of β-lap for the indicated times, and were subjected to Western blot analysis using anti-AIF, -Endo G, and -Nucleoporin p62 antibodies. Nucleoporin p62 was used as a nuclear marker protein. The data are representative a typical experiment conducted three times. The data are representative of a typical experiment conducted three times with similar results. (B) Representative confocal images showing translocation of AIF and Endo G to the nucleus, and nuclear condensation, at the indicated times after treatment with 5 µM of β-lap. Nuclear translocation of AIF and Endo G is demonstrated by overlap of AIF or Endo G (green) and nuclear staining (red), resulting in a yellow color. The data are representative of a typical experiment conducted three times. (C) NQO1^+^-MDA-MB-231 cells transfected with AIF or Endo G siRNA were treated with 5 µM of β-lap. Cells were allowed to grow for 10 to 14 days and were stained with 0.5% crystal violet and scored for colony formation. Results from three independent experiments are expressed as means ± SEM. *Significant difference between si-cont RNA- and si-AIF RNA-transfected cells after β-lap treatment, at p<0.05. **Significant difference between si-cont RNA- and si-Endo G RNA-transfected cells after β-lap treatment, at p<0.05. (D) NQO1^+^-MDA-MB-231 cells transfected with AIF or Endo G siRNA were treated with 5 µM of β-lap for 18 h. Cells were stained with PI and the percentage of cells with a sub-G1 DNA content was measured via flow cytometric analysis. Results from three independent experiments are expressed as the means ± SEM. *Significant difference between si-cont RNA- and si-AIF RNA-transfected cells after β-lap treatment, at p<0.05. **Significant difference between si-cont RNA- and si-Endo G RNA-transfected cells after β-lap treatment, at p<0.05.

AIF and Endo G have been known to play key roles in induction of a caspase-independent apoptotic cell death [10]. Therefore, we next examined whether they are involved in β-lap-induced apoptotic cell death. Subcellular fractionation showed that the treatment of NQO1^+^-MDA-MB-231 cells with β-lap dramatically induced the translocation of AIF and Endo G to the nucleus (Fig. 2A). Confocal microscopy also clearly showed that β-lap treatment caused translocation of AIF and Endo G to nucleus, and induced nuclear condensation (Fig. 2B). Moreover, siRNAs targeting AIF and Endo G effectively attenuated the clonogenic and apoptotic cell death caused by β-lap treatment (Fig. 2C and D).

These results suggest that nuclear translocation of AIF and Endo G is required for the induction of apoptotic cell death in NQO1^+^-MDA-MB-231 cells after β-lap treatment.

### β-lap induces generation and translocation of cleaved Bax to mitochondria

**Figure 3:**
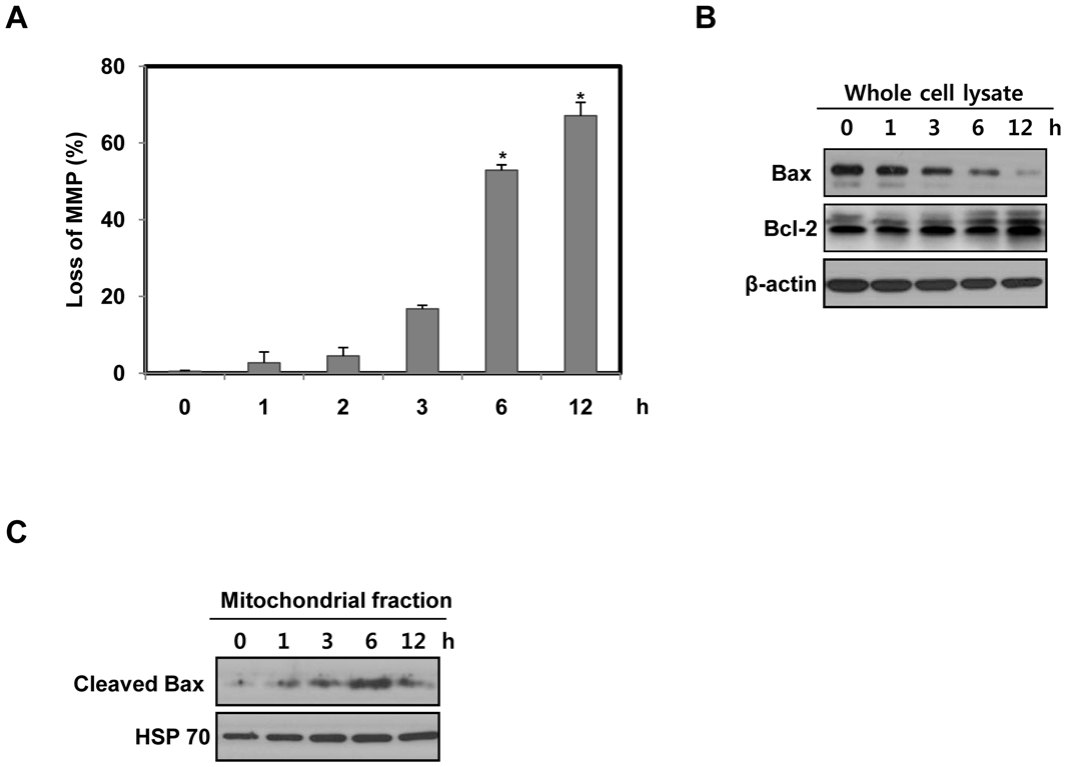
β-lap causes Bax cleavage and translocation of cleaved Bax to mitochondria. (A) NQO1^+^-MDA-MB-231 cells were treated with 5 µM of β-lap for the indicated times. After 12 h, cells were loaded with 30 nM of DiOC_6_(3) for a further 30 min of the treatments. After removal of medium, the concentration of retained DiOC_6_(3) was measured by flow cytometry. Results from three independent experiments are expressed as means ± SEMs. *Significant difference between control and β-lap-treated cells at p<0.05. (B) NQO1^+^-MDA-MB-231 cells were treated with 5 µM of β-lap for the indicated times. Cell lysates were subjected to Western blot analysis using anti-Bax, -Bcl-2 and -β-actin antibodies. β-actin was used as a loading control. The data represent a typical experiment conducted three times with similar results. (C) NQO1^+^-MDA-MB-231 cells were treated with 5 µM of β-lap for the indicated times. Mitochondrial fractions were prepared and subjected to Western blot analysis using anti-Bax and -mitochondrial HSP70 antibodies. Mitochondrial HSP70 was used as a mitochondrial marker protein. The data are representative a typical experiment conducted three times.

To assess the role of the mitochondrial pathway in induction of apoptosis after β-lap treatment, we first investigated the changes in mitochondrial membrane potential, and the expression levels of Bcl-2 family proteins in NQO1^+^-MDA-MB-231 cells after β-lap treatment. As shown in Figure 3A, β-lap treatment significantly increased the disruption of mitochondrial membrane potential in a time-dependent manner. Simultaneously, Bax level markedly decreased, whereas Bcl-2 level did not alter in response to β-lap treatment (Fig. 3B).

Previously, Bax has been reported to be cleaved to an 18 kDa fragment from 21 kDa native form in response to diverse stimuli [15], [16]. As translocation of cleaved Bax from the cytosol to mitochondria expedited a decline in mitochondrial membrane potential and subsequent release of apoptogenic proteins from mitochondria [15], [16], we next determined whether β-lap treatment induces mitochondrial translocation of cleaved Bax. Interestingly, Figure 3C shows that β-lap treatment effectively augmented the level of cleaved Bax within mitochondrial fractions, in agreement with the observed changes in mitochondrial membrane potential. However, there were no indications of presence of 18-kDa cleaved form of Bax in whole cell lysates after β-lap treatment (Fig. 3B). Since a cathepsin-like protease has been reported to be involved in the rapid degradation of p18 kDa cleaved from of Bax in the cytosol [14], cleaved Bax is thought to be readily degraded in the cytosol after β-lap treatment.

These results indicate that an alteration in mitochondrial membrane potential mediated by intracellular redistribution of cleaved Bax is involved in β-lap-induced cell death.

### JNK activation is required for the induction of mitochondria-mediated apoptotic cell death caused by β-lap

**Figure 4:**
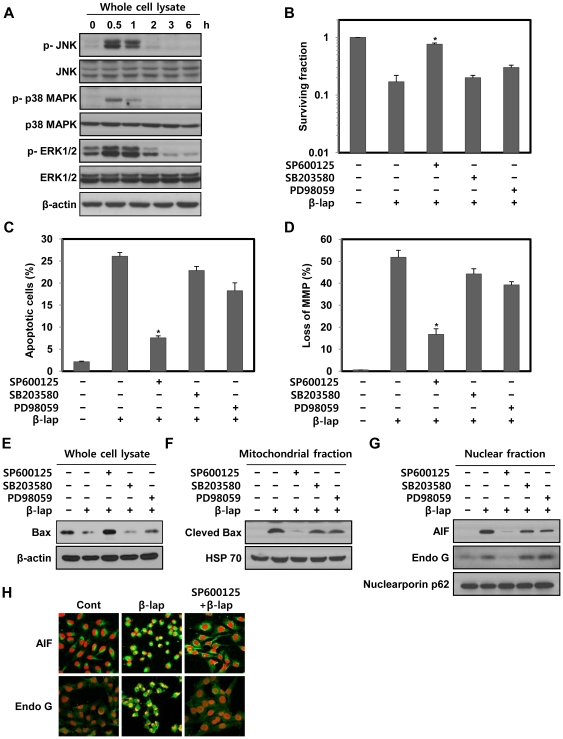
β-lap activates MAPKs in NQO1^+^-MDA-MB-231 cells. (A) NQO1^+^-MDA-MB-231 cells were treated with 5 µM of β-lap for the indicated times. Cell lysates were subjected to Western blot analysis with anti-phospho-ERK1/2, -ERK1/2, -phospho-JNK1/2, -JNK1/2, -phsopho-p38 MAPK, -p38 MAPK and β-actin antibodies. β-actin was used as a loading control. The data represent a typical experiment conducted three times with similar results. (B) NQO1^+^-MDA-MB-231 cells were treated with 5 µM of β-lap in the presence or absence of PD98059 (30 µM), SP600125 (30 µM) or SB203580 (30 µM). Cells were allowed to grow for 10 to 14 days and were stained with 0.5% crystal violet and scored for colony formation. Results from three independent experiments are expressed as means ± SEM. *Significant difference between cells in the presence or absence of SP600125 after β-lap treatment, at p<0.05. (C) NQO1^+^-MDA-MB-231 cells were treated with 5 µM of β-lap for 18 h in the presence or absence of PD98059 (30 µM), SP600125 (30 µM) or SB203580 (30 µM). Cells were stained with PI and the percentage of cells with a sub-G1 DNA content was measured via flow cytometric analysis. Results from three independent experiments are expressed as the means ± SEM. *Significant difference between the cells in the presence or absence of SP600125 after β-lap treatment at p<0.05. (D) NQO1^+^-MDA-MB-231 cells were treated with 5 µM of β-lap for 6 h in the presence or absence of PD98059 (30 µM), SP600125 (30 µM) or SB203580 (30 µM). Cells were loaded with 30 nM of DiOC_6_(3) for a further 30 min of the treatments. After removal of medium, the concentration of retained DiOC_6_(3) was measured by flow cytometry. Results from three independent experiments are expressed as means ± SEMs. *Significant difference between the cells in the presence or absence of SP600125 after β-lap treatment at p<0.05. (E) NQO1^+^-MDA-MB-231 cells were treated with 5 µM of β-lap for 6 h in the presence or absence of PD98059 (30 µM), SP600125 (30 µM) or SB203580 (30 µM). Cell lysates were subjected to Western blot analysis using anti-Bax, -Bcl-2 and -β-actin antibodies. β-actin was used as a loading control. The data represent a typical experiment conducted three times with similar results. (F) NQO1^+^-MDA-MB-231 cells were treated with 5 µM of β-lap for 6 h in the presence or absence of PD98059 (30 µM), SP600125 (30 µM) or SB203580 (30 µM). Mitochondrial fractions were prepared and subjected to Western blot analysis using anti-Bax and -mitochondrial HSP70 antibodies. Mitochondrial HSP70 was used as a mitochondrial marker protein. The data are representative a typical experiment conducted three times. (G) NQO1^+^-MDA-MB-231 cells were treated with 5 µM of β-lap for 6 h in the presence or absence of PD98059 (30 µM), SP600125 (30 µM) or SB203580 (30 µM). Nuclear fractions were prepared and subjected to Western blot analysis using anti-AIF, -Endo G, and -Nucleoporin p62 antibodies. Nucleoporin p62 was used as a nuclear marker protein. The data are representative a typical experiment conducted three times. The data are representative of a typical experiment conducted three times with similar results. (H) Representative confocal images showing translocation of AIF and Endo G to the nucleus, and nuclear condensation, at the indicated times after treatment with 5 µM of β-lap for 6 h in the presence or absence of SP600125 (30 µM). Nuclear translocation of AIF and Endo G is demonstrated by overlap of AIF or Endo G (green) and nuclear staining (red), resulting in a yellow color. The data are representative of a typical experiment conducted three times.

In order to elucidate the involvement of MAPKs in β-lap-induced apoptotic cell death, we first analyzed the kinetics of MAPK activation following β-lap treatment in NQO1^+^-MDA-MB-231 cells. Although no changes in the total cellular levels of JNK, p38 MAPK, and ERK were evident, β-lap treatment rapidly increased the levels of phosphorylated form of JNK, p38 MAPK, and ERK within 30 min after β-lap treatment (Fig. 4A).

To identify the specific MAPK involved in β-lap-induced cell death, we pretreated the cells with SP600125, SB203580, and PD98059; inhibitors of JNK, p38 MAPK, and MEK, respectively, prior to β-lap treatment. Interestingly, SP600125 selectively and effectively reduced β-lap-induced clonogenic and apoptotic cell death (Fig. 4B and C). Pretreatment with PD98059 or SB203580 slightly attenuated β-lap-mediated cellular responses in this regard (Fig. 4B and C).

We next investigated the roles of MAPKs on mitochondria-mediated cell death caused by β-lap treatment. Figures 4D, E, F and G show that SP600125 was fairly more effective than either SB203580 or PD98059 in suppressing all of β-lap-induced disruption of mitochondrial membrane potential, Bax cleavage, mitochondria translocation of cleaved Bax, and nuclear translocation of AIF and Endo G. Confocal microscopy also clearly showed that SP600125 efficiently blocked the nuclear translocation of AIF and Endo G induced by β-lap treatment (Fig. 4H).

**Figure 5:**
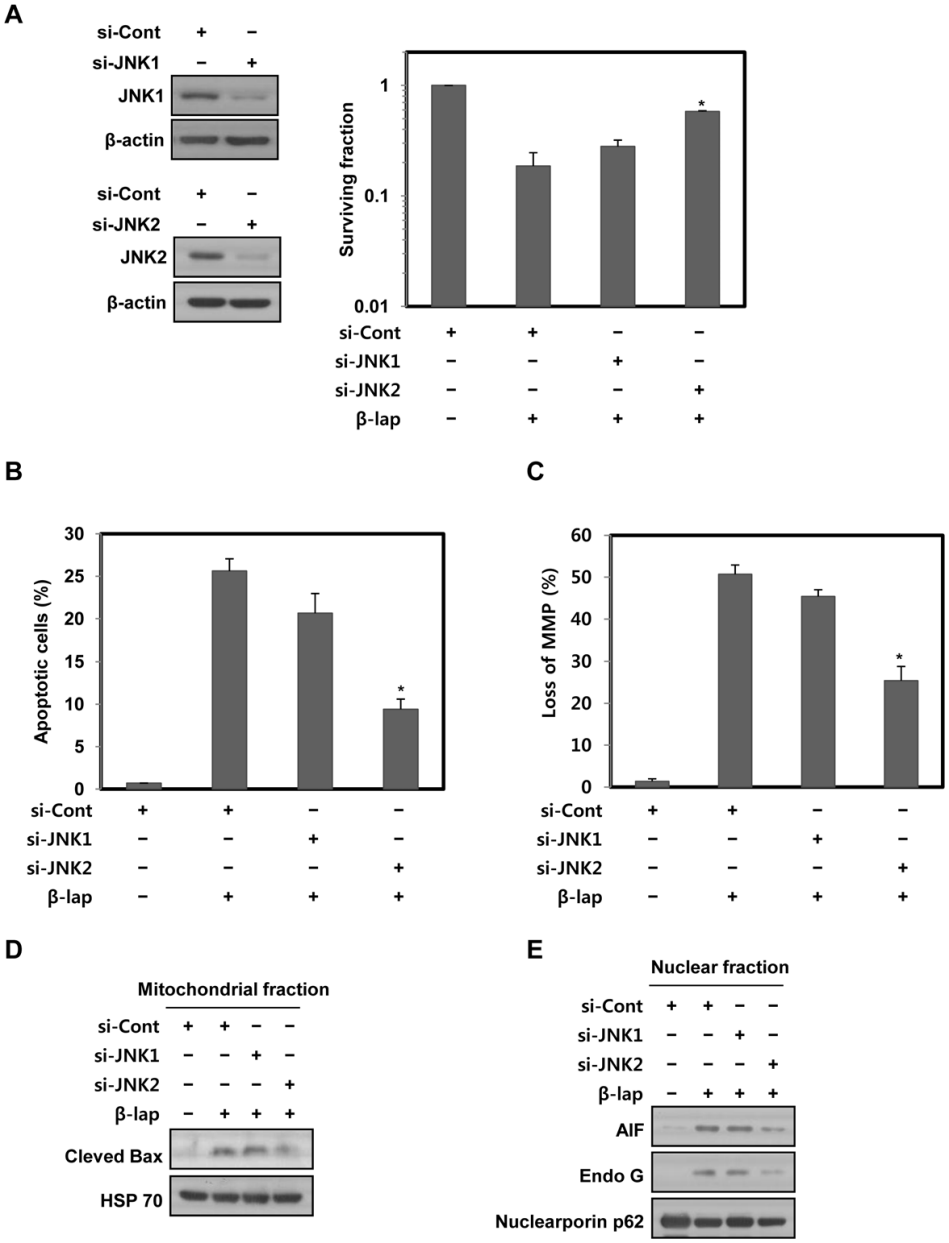
The activity of JNK2 is necessarily required for mitochondria-mediated cell death caused by β-lap. (A) NQO1^+^-MDA-MB-231 cells transfected with JNK1 or JNK2 siRNA were treated with 5 µM of β-lap. Cells were allowed to grow for 10 to 14 days and were stained with 0.5% crystal violet and scored for colony formation. Results from three independent experiments are expressed as means ± SEM. *Significant difference between si-cont RNA- and si-JNK2 RNA-transfected cells after β-lap treatment, at p<0.05. (B) NQO1^+^-MDA-MB-231 cells transfected with JNK1 or JNK2 siRNA were treated with 5 µM of β-lap for 18 h. Cells were stained with PI and the percentage of cells with a sub-G1 DNA content was measured via flow cytometric analysis. Results from three independent experiments are expressed as the means ± SEM. *Significant difference between si-cont RNA- and si-JNK2 RNA-transfected cells after β-lap treatment, at p<0.05. (C) NQO1^+^-MDA-MB-231 cells transfected with JNK1 or JNK2 siRNA were treated with 5 µM of β-lap for 6 h. Cells were loaded with 30 nM of DiOC_6_(3) for a further 30 min of the treatments. After removal of medium, the concentration of retained DiOC_6_(3) was measured by flow cytometry. Results from three independent experiments are expressed as means ± SEMs. *Significant difference between si-cont RNA- and si-JNK2 RNA-transfected cells after β-lap treatment, at p<0.05. (D) NQO1^+^-MDA-MB-231 cells transfected with JNK1 or JNK2 siRNA were treated with 5 µM of β-lap for 6 h. Mitochondrial fractions were prepared and subjected to Western blot analysis using anti-Bax and -mitochondrial HSP70 antibodies. Mitochondrial HSP70 was used as a mitochondrial marker protein. The data are representative a typical experiment conducted three times. (E) NQO1^+^-MDA-MB-231 cells transfected with JNK1 or JNK2 siRNA were treated with 5 µM of β-lap for 6 h. Nuclear fractions were prepared and subjected to Western blot analysis using anti-AIF, -Endo G, and -Nucleoporin p62 antibodies. Nucleoporin p62 was used as a nuclear marker protein. The data are representative a typical experiment conducted three times. The data are representative of a typical experiment conducted three times with similar results.

Because it was obvious that JNK played a prominent role in β-lap-induced mitochondrial apoptosis, we further determined which JNK isoform was required for the β-lap-induced cell death using siRNA targeting JNK1 and JNK2. As shown in Figures 5A, B and C, siRNA targeting JNK2 efficiently attenuated β-lap-induced clonogenic and apoptotic cell death, and dissipation of mitochondrial membrane potential, whereas siRNA targeting JNK1 only slightly reduced them. Furthermore, siRNA targeting JNK2 is significantly more effective than siRNA targeting JNK1 in blocking all of β-lap-induced mitochondrial translocation of cleaved Bax, and nuclear translocation of AIF and Endo G (Fig. 5D and E). These results clearly indicate that JNK2 is a prominent mediator of β-lap-induced mitochondrial apoptosis in NQO1^+^-MDA-MB-231 cells.

### ER stress induced by β-lap is required for the activation of JNK

**Figure 6:**
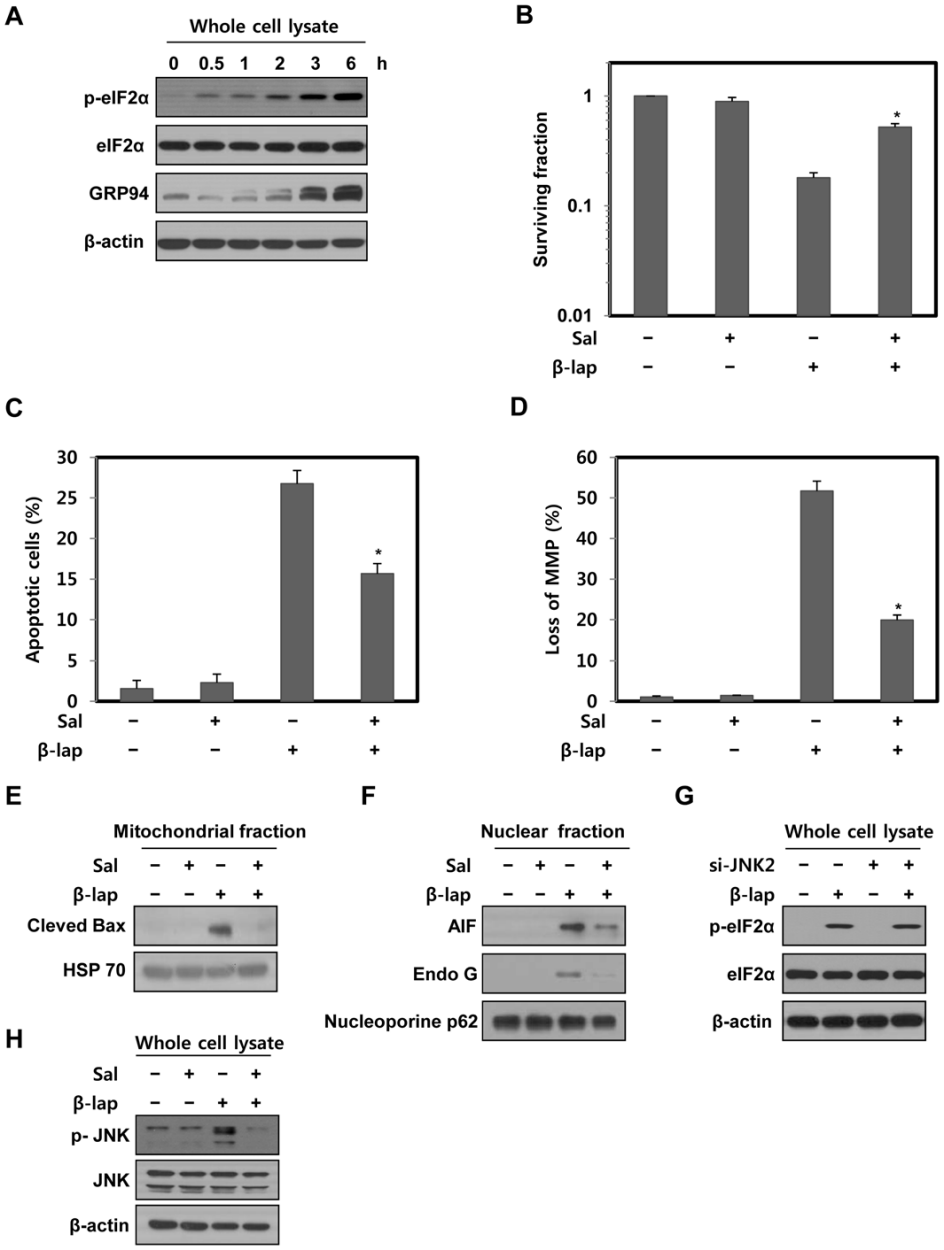
The induction of ER stress caused by β-lap is required for JNK activation. (A) NQO1^+^-MDA-MB-231 cells were treated with 5 µM of β-lap for the indicated times. Cell lysates were subjected to Western blot analysis with anti-phospho-eIF2α, -eIF2α, -GRP94 and β-actin antibodies. β-actin was used as a loading control. The data represent a typical experiment conducted three times with similar results. (B) NQO1^+^-MDA-MB-231 cells were treated with 5 µM of β-lap in the presence or absence of Salubrinal (30 µM). Cells were allowed to grow for 10 to 14 days and were stained with 0.5% crystal violet and scored for colony formation. Results from three independent experiments are expressed as means ± SEM. *Significant difference between cells in the presence or absence of Salubrinal after β-lap treatment, at p<0.05. (C) NQO1^+^-MDA-MB-231 cells were treated with 5 µM of β-lap for 18 h in the presence or absence of Salubrinal (30 µM). Cells were stained with PI and the percentage of cells with a sub-G1 DNA content was measured via flow cytometric analysis. Results from three independent experiments are expressed as the means ± SEM. *Significant difference between the cells in the presence or absence of Salubrinal after β-lap treatment at p<0.05. (D) NQO1^+^-MDA-MB-231 cells were treated with 5 µM of β-lap for 6 h in the presence or absence of Salubrinal (30 µM). Cells were loaded with 30 nM of DiOC_6_(3) for a further 30 min of the treatments. After removal of medium, the concentration of retained DiOC_6_(3) was measured by flow cytometry. Results from three independent experiments are expressed as means ± SEMs. *Significant difference between the cells in the presence or absence of Salubirnal after β-lap treatment at p<0.05. (E) NQO1^+^-MDA-MB-231 cells were treated with 5 µM of β-lap for 6 h in the presence or absence of Salubrinal (30 µM). Mitochondrial fractions were prepared and subjected to Western blot analysis using anti-Bax and -mitochondrial HSP70 antibodies. Mitochondrial HSP70 was used as a mitochondrial marker protein. The data are representative a typical experiment conducted three times. (F) NQO1^+^-MDA-MB-231 cells were treated with 5 µM of β-lap for 6 h in the presence or absence of Salubrinal (30 µM). Nuclear fractions were prepared and subjected to Western blot analysis using anti-AIF, -Endo G, and -Nucleoporin p62 antibodies. Nucleoporin p62 was used as a nuclear marker protein. The data are representative a typical experiment conducted three times. The data are representative of a typical experiment conducted three times with similar results. (G) NQO1^+^-MDA-MB-231 cells transfected with JNK2 siRNA were treated with 5 µM of β-lap for 3 h. Cell lysates were subjected to Western blot analysis with anti-phospho-eIF2α, -eIF2α and -β-actin antibodies. β-actin was used as a loading control. The data represent a typical experiment conducted three times with similar results. (H) NQO1^+^-MDA-MB-231 cells were treated with 5 µM of β-lap for 30 min in the presence or absence of Salubrinal (30 µM). Cell lysates were subjected to Western blot analysis with anti-phospho-JNK1/2, -JNK1/2, and -β-actin antibodies. β-actin was used as a loading control. The data represent a typical experiment conducted three times with similar results.

ER stress has been reported to trigger apoptotic cell death [23]–[29]. Therefore, we assessed whether β-lap induces ER stress in NQO1^+^-MDA-MB-231 cells. As shown in Figure 6A, β-lap treatment significantly increased the phsophorylation of eIF2α and the expression of GRP94 for 6 h. To further examine the involvement of ER stress in β-lap-induced cell death, we pretreated NQO1^+^-MDA-MB-231 cells with Salubrinal (Sal), an ER stress inhibitor. As shown in Figures 6B and C, pretreatment with Sal effectively attenuated β-lap-induced clonogenic and apoptotic cell death. We next investigated the roles of ER stress on mitochondria-mediated cell death caused by β-lap treatment. Figures 6D, E and F show that pretreatment with Sal significantly suppressed β-lap-induced disruption of mitochondrial membrane potential, mitochondrial translocation of cleaved Bax and the nuclear translocation of AIF and Endo G. These results clearly indicate that ER stress is important for mitochondria-mediated cell death caused by β-lap treatment.

Because ER stress has been known to be one of the main causes of JNK activation [23]–[29], we further assessed the relationship between ER stress and JNK activation using a pharmacological inhibitor of ER stress and JNK2 siRNA. As shown in Figures 6G and H, siRNA targeting JNK2 did not attenuate the phosphorylation of eIF2α caused by β-lap treatment, whereas pretreatment with Sal significantly prevented the activation of JNK. These findings suggest that ER stress is strictly necessary for the activation of JNK after β-lap treatment.

## Discussion

β-lap, a novel anticancer drug, is bioreductive agent which is reduced in cells by the reductive enzyme, NQO1 (1–6). The reduction of β-lap induces Ca^2+^ dependent hyperactivation of Poly (ADP-Ribose) polymerase-1 (PARP-1) and DNA damage, which subsequently contributes to apoptotic or necrotic cell death [30]–[35]. In the present work, β-lap preferentially induced clonogenic and apoptotic cell death in NQO1^+^-MDA-MB-231 cells compared to NQO1^−^-MDA-MB-231 cells (Fig. 1). These results are consistent with previous data on the role played by NOQ1 in mediation of β-lap toxicity [30]–[34], [36]. Because NQO1 is abundantly expressed in a variety of cancer tissues, it may be surmised that cancer tissues can be damaged selectively by β-lap. However, the molecular signaling pathways underlying the therapeutic effects of β-lap on solid cancers have not yet been clearly understood. In the present study, we demonstrate that the nuclear translocation of AIF and Endo G is critical for β-lap-induced caspase-independent cell death. We also show that JNK activation triggered by ER stress is essential for mitochondria-mediated cell death caused by β-lap.

Many anti-cancer drugs have been suggested to induce apoptosis of cancer cells via mitochondrial dysfunction linked to caspase-dependent/independent pathways [37], [38]. Although β-lap has been previously reported to induce apoptotic cell death in a caspase-dependent manner [39], [40], the apoptotic process caused by β-lap in the present work, appears to be caspase-independent, because a broad spectrum caspase inhibitor failed to suppress β-lap-induced apoptosis (Fig. 1).

AIF and Endo G are well known to be involved in a caspase-independent apoptotic pathway [7], [10], [11]. Death stimuli accelerate the release of AIF and Endo G from mitochondria, and then these proteins are translocated to the nucleus, where they initiate nuclear condensation and DNA fragmentation [7], [10], [11]. In agreement with these reports, we observed that AIF and Endo G are translocated to the nucleus, and the activities of these proteins are also required for β-lap-induced cell death (Fig. 2).

Bcl-2 family plays key roles in the regulation of mitochondria-mediated cell death [6]. The anti-apoptotic Bcl-2 family proteins including Bcl-2, Bcl-xL and Mcl-1 are involved in cell survival [6]. Recent reports demonstrated that STAT3 (signal transducer and activator of transcription 3) increases the transcription and subsequent expression of Bcl-2, Bcl-xL and Mcl-1 [41]–[43]. As this pro-oncogenic transcription factor is constitutively activated in many types of cancer, it is highly likely that STAT3 also plays an important role in cell proliferation, cell survival and tumor transformation [42]–[44]. Moreover, STAT3 has been suggested to be involved in IL-6-induced resistance to β-lap in prostatic cancer cells [41]. In response to diverse stimuli, Bax is activated, translocated to the outer mitochondrial membrane, and oligomerized, resulting in the disruption of mitochondrial integrity and release of mitochondrial apoptogenic proteins to the cytosol [12]–[14]. In particular, N-terminal cleavage of native Bax (21 kDa) into an 18 kDa form by calpain has been reported to occur in cancer cells treated with a variety of chemotherapeutic drugs [15], [16]. This cleaved form of Bax is more potent than the native form for disruption of mitochondrial membrane potential and induction of apoptotic cell death. In agreement with these reports, we observed that β-lap leads to Bax cleavage without alterations in the protein level of Bcl-2. We also found that the cleaved form of Bax is translocated to mitochondria upon treatment with β-lap (Fig. 3).

It has already been suggested that β-lap activates MAPKs in human cancer cells [45], [46]. In agreement with these reports, we observed that all MAPKs are activated soon after β-lap treatment (Fig. 4). In particular, inhibition of JNK by a specific inhibitor effectively attenuates all of β-lap-induced clonogenic, apoptotic cell death, disruption of mitochondrial membrane potential, cleavage of Bax, mitochondrial translocation of cleaved Bax, and nuclear translocation of AIF and Endo G. However, inhibition of p38 MAPK or ERK slightly inhibits these events triggered by β-lap, indicating that the activations of both p38 MAPK and ERK are weakly associated with β-lap-induced cell death. These observations are consistent with a previous report that JNK plays a critical role during β-lap-induced apoptosis in human cancer cells [46]. Although the cited report did not show the downstream targets of JNK during β-lap-induced apoptosis, our results in the present study clearly indicated that JNK activation is required for activation of the mitochondria-mediated cell death pathway in response to β-lap treatment. Additionally, since calpain participating in the proteolytic cleavage of Bax has been reported to be activated indirectly by JNK [47], [48], and β-lap was also suggested to activate calpain [1], [2], [5], further studies are required to clarify the role of JNK in calpain-mediated Bax cleavage occurring in response to β-lap. Furthermore, we found that inhibition of JNK2 is more effective than that of JNK1 in blocking all of β-lap-induced apoptosis, loss of mitochondrial membrane potential, mitochondrial translocation of cleaved Bax, and nuclear translocation of AIF and Endo G (Fig. 5). These findings are consistent with the results of a previous study that JNK2 activity was required for mitochondria-mediated apoptosis in response to liver ischemia [49]. Furthermore, because JNK1 plays a crucial role in controlling systemic glucose and lipid metabolism involved in energy expenditure [50], and was proposed as a candidate protein kinase that can phosphorylate Zfra (zinc finger-like protein that regulates apoptosis) known to participate in mitochondria-mediated apoptosis [51], there is a possiblity that JNK1 can partially affect β-lap-induced mitochondrial apoptosis via regulation of metabolic pathways or Zfra phosphorylation.

It is noteworthy that β-lap-induced activation of JNK started to increase within 30 min, and lasted for 1 h and then subsequently decreased (Fig. 4). This reduction of JNK activity in 1 h following β-lap treatment may be due to the activation of phosphatases, such as a MAPK phosphatase 2 (MKP2) and -7 (MKP7), which have been reported to regulate the intracellular MAPK signaling pathways [52]-[54]. Therefore, because there is a reasonable possibility that JNK activity can be regulated by a negative feedback loop between JNK and these MAPK phosphatases in response to β-lap, additional studies are needed to define the precise role played by MKP2 or MKP7 in β-lap-treated cells.

It has been clearly demonstrated that excessive ER stress can lead to apoptotic cell death [23]–[26]. Consistent with these reports, we found that β-lap induces the phosphorylation of eIF2α, and raises the expression level of GRP94 in NQO1^+^-MDA-MB-231 cells. We also observed that inhibition of ER stress by pretreatment with Sal, an ER stress inhibitor, efficiently attenuates mitochondria-mediated cell death caused by β-lap. These results suggested that the cell death induced by β-lap treatment is mainly due to induction of ER stress. The activation of JNK by ER stress has been attributed to activation of IRE1/TRAF2/ASK1 pathway [23]–[26]. In response to ER stress, IRE1 is activated by autophosphorylation, and then recruits TRAF2 (tumor necrosis factor receptor-associated factor 2), which subsequently activates ASK1 leading to JNK activation. In agreement with previous reports, our results showed that the suppression of ER stress by Sal effectively blocked JNK activation and subsequent apoptotic cell death caused by β-lap, whereas the inhibition of JNK by pretreatment with JNK2 siRNA did not attenuate β-lap-induced ER stress (Fig. 6). These results suggested that JNK activation acts on downstream of the ER stress to induce mitochondria-mediated cell death in response to β-lap treatment.

**Figure 7:**
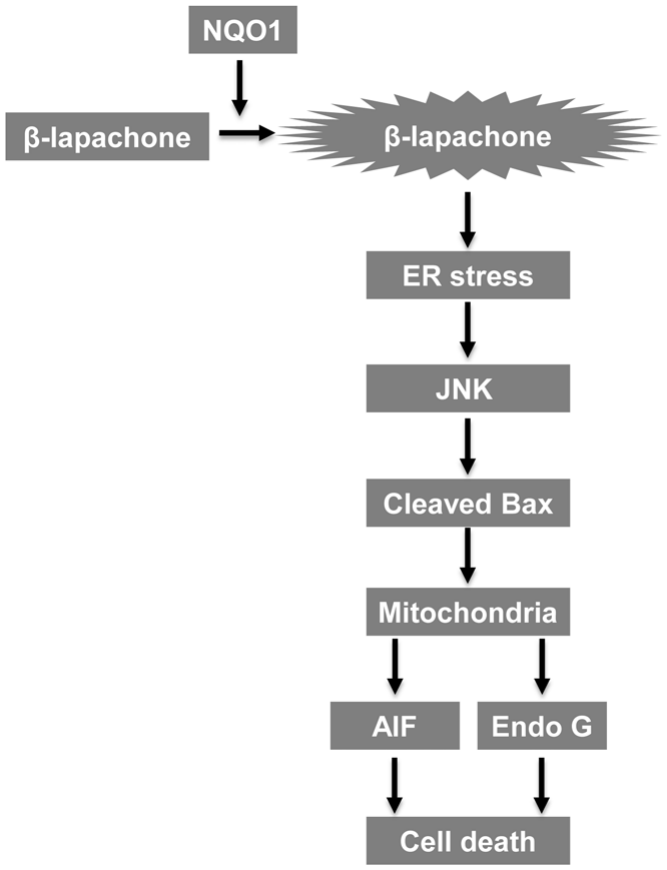
Schematic model of mitochondria-mediated cell death triggered by β-lap. β-lap induces mitochondrial apoptotic cell death in a NQO1-dependent manner. The induction of ER stress by β-lap is substantially required for JNK activation which triggers mitochondria-mediated cell death pathway. In detail, JNK activation induces cleavage of Bax and mitochondrial translocation of cleaved Bax, which causes disruption of mitochondrial membrane potential, and subsequent nuclear translocation of AIF and Endo G. Understanding these signaling pathways that we elucidated in this study may provide useful insights into improvement in therapeutic efficacy of the design of NQO1-targeted cancer therapies.

In conclusion, in the present study, we demonstrate here that β-lap significantly induces apoptotic cell death in a NQO1-dependent manner, and also suggest that ER stress-induced JNK activation is a critical requirement for mitochondria-mediated cell death caused by β-lap (Fig. 7). Better insights into the signaling pathways underlying the anti-cancer activity of β-lap will lead to advancement in treatment strategies of NQO1-directed cancer therapy using the novel drug.

## Materials and Methods

### Reagents

β-lap was purchased from Biomol (Plymouth Meeting, PA), and was dissolved in DMSO. Antibodies against p38 MAPK, JNK2, JNK1/2, ERK1/2, Bax, Bcl2, AIF and Nucleoporin p62 were purchased from Santa Cruz Biotechnology, Inc. (Santa Cruz, CA). An antibody against mitochondrial heat shock protein 70 (HSP70) was the product of Affinity Bioreagents (Golden, CO). Anti-β-actin, -rabbit IgG, and -mouse IgG were purchased from Sigma (St. Lous, MO). Antibodies against phospho-p38 MAPK (Thr180/Tyr182), phospho-JNK1/2 (Thr183/Tyr185), phospho-ERK1/2 (Thr202/Tyr204), phosphor-eIF2α (Ser51), and eIF2α were obtained from Cell Signaling Technology (Beverly, MA). Inhibitors specific to JNK (SP600125), MEK/ERK (PD98059), p38 MAPK (SB203580), caspases (z-VAD-fmk) and ER stress (Salubrinal) were purchased from Calbiochem (San Diego, CA).

### Cell culture

Parental NQO1^−^-MDA-MB-231 human breast cancer cells, which are NQO1-deficient, and NQO1^+^-MDA-MB-231 human breast cancer cells, which are stably transfected with NQO1, were obtained from Dr. David Boothman, University of Texas Southwestern Medical Center, Dallas, TX. Cells were cultured in RPMI 1640 medium (Gibco BRL, Grand Island, NY) supplemented with 10% (v/v) bovine calf serum (Gibco BRL), penicillin (50 units/ml), and streptomycin (50 µg/ml), in a 37°C incubator under a mixture of 95% air and 5% CO2 (both v/v).

### Small interfering RNA transfection

RNA interference with siRNAs was carried out using double-stranded RNA molecules. AIF (5′-GCA AGU UAC UUA UCA AGC UTT-3′) and JNK2 (5′-CUG UAA CUG UUG AGA UGU ATT-3′) siRNAs was purchased from Bioneer Corporation (Daejeon, Korea). Endo G (5′-GGA ACA ACC UGG AGA AAU ATT-3′) and JNK1 (5′- GAC CUA AAU AUG CUG GAU ATT-3′) siRNAs were the products of Samchully Pharm (Seoul, Korea). An unrelated control siRNA (5′-CCA CTA CCT GAG CAC CCA G-3′) that targeted green fluorescent protein DNA sequence was used as a control. For transfection, cells were seeded on 60 mm dishes and transfected at 30% confluency with the siRNA duplexes (100 nM), using Lipofectamine 2000 (Invitrogen, Carlsbad, CA) in accordance with the manufacturer's instructions. Assays were performed 24 h after transfection.

### Quantification of clonogenic death

Various numbers of cells were plated on 60 mm dishes and treated with a range of doses of β-lap at 0 to 10 µM, or 5 µM of β-lap, and followed by incubation for 14 days at 37°C in 5% CO2 incubator. Prior to counting colonies, the culture medium was decanted and the cells were fixed in 95% methanol and stained with 0.5% crystal violet, and the numbers of colonies (>50 cells) from triplicate dishes were counted. Mean colony numbers relative to unirradiated colony numbers were plotted.

### Quantification of cell death

Cells were collected by trypsinization, washed 2 times with PBS, resuspended in 1 ml PBS containing 0.1% Triton X-100, 0.1 mM EDTA, 10 mg/ml DNase-free RNase A, and 2 mg/ml propidium iodide (PI), and incubated for 1 h in the dark at 37°C. Apoptotic cells were detected on a flow cytometer using a FACSCalibur (Becton Dickinson, San Jose, CA). Apoptosis was measured as the percentage of sub-G1 cell population.

### Western blot analysis

Cells were treated with lysis buffer [40 mmol/L Tris-Cl (pH 8.0), 120 mmol/L NaCl, and 0.1% NP40] supplemented with protease inhibitors, then centrifuged for 15 min at 12,000× g. Proteins were separated via SDS-PAGE and transferred to nitrocellulose membranes (Bio-Rad, Hercules, CA). The membranes were blocked with 5% nonfat dry milk in Tris-buffered saline and subsequently incubated for 1 h with primary antibodies at room temperature. Blots were developed with peroxidase-conjugated secondary antibody, and the proteins were visualized via enhanced chemiluminescence (Amersham Biosciences, Piscataway, NJ) according to the manufacturer's recommendations.

### Confocal microscopy

β-lap-treated cells were washed twice in ice-cold PBS prior to fixation in ice-cold methanol. After blocking with 2% (w/v) bovine serum albumin in PBS containing 0.2% (v/v) Triton X-100, cells were incubated for 1 h with primary antibodies against AIF and Endo G. Cells were then washed three times in blocking solution and incubated for an additional hour with secondary antibody conjugated to FITC (Molecular Probes). Nuclei were then stained with PI (Sigma) for 10 minutes. After three further washes with PBS, coverslips were mounted onto microscope slides using the ProLong Antifade mounting reagent (Molecular Probes). Slides were viewed using a confocal laser-scanning microscope (Nikon TE-2000E, Tokyo, Japan).

### Measurement of mitochondrial membrane potential

Cells were incubated for 30 min in 30 nM of DiOC_6_(3) (Molecular Probes, Eugene, OR) at 37°C, harvested via trypsinization, and washed three times with cold PBS. Mitochondrial membrane potential was determined by flow cytometry.

### Preparation of mitochondrial and nuclear fractions

Cells were collected and washed twice in ice-cold PBS, resuspended in isotonic homogenization buffer [250 mM sucrose, 10 mM KCl, 1.5 mM MgCl_2_, 1 mM Na-EDTA, 1 mM dithiothreitol, 0.1 mM phenylmethylsulfonylfluoride, 10 mM Tris-HCl (pH 7.4)], incubated on ice for 20 min, and homogenized using a Dounce glass homogenizer fitted with a loose pestle (Wheaton, Millville, NJ) (70 strokes). Cell homogenates were spun at 30× g to remove any unbroken cells. Supernatants were then respun for 10 min at 750× g to separate nuclear and mitochondrial fractions. Each nuclear fraction (a pellet) was washed three times in homogenization buffer, and resuspended in lysis buffer [50 mM Tris-HCl (pH 7.5), 150 mM NaCl, 1% (v/v) NP40, 0.5% (w/v) sodium deoxycholate] containing protease inhibitors, prior to Western blot analysis. After pelleting of the nuclear fraction, the supernatant was further subjected to 30 min of centrifugation at 14,000× g to pellet a mitochondria-rich fraction. Pellets were washed once in homogenization buffer, and then resuspended in lysis buffer [150 mM NaCl, 50 mM Tris-HCl (pH 7.5), 1% (v/v) NP40, 0.25% (w/v) sodium deoxycholate, and 1 mM EGTA], with protease inhibitors, prior to Western blot analysis.

### Statistical analysis

All data presented are representative of at least three separate experiments. Comparisons among groups were analyzed using Student's t-test (SPSS Statistics version 17.0, Chicago, IL). p values <0.05 were considered to be significant.
